# Metabolic changes during malignant transformation in primary cells of oral lichen planus: Succinate accumulation and tumour suppression

**DOI:** 10.1111/jcmm.14376

**Published:** 2019-12-02

**Authors:** Qiaozhen Yang, Hongying Sun, Xiaxia Wang, Xuedi Yu, Jie Zhang, Bin Guo, Saiyin Hexige

**Affiliations:** ^1^ Department of Stomatology Huashan Hospital, Fudan University Shanghai China; ^2^ School of Life Sciences Fudan University Shanghai China

**Keywords:** HIF‐1α, MEG3, oral lichen planus, oral squamous cell carcinoma, succinate

## Abstract

Oral squamous cell carcinoma (OSCC) is usually diagnosed at late stages, which leads to high morbidity. There are evidence that chronic inflammation (eg oral lichen planus [OLP]) was a risk factor of OSCC, but often misdiagnosed or ignored until invasion and metastasis. By applying precision medicine, the molecular microenvironment variations and relevant biomarkers for the malignant transformation from OLP to OSCC can be fully investigated. Several studies pointed out that the metabolic pathway were suppressed in OSCC. However, it remains unclear how the systemic profile of the metabolites change during the malignant transformation. In this study, we examined and compared the mucosa samples from 11 healthy individuals, 10 OLP patients and 21 OSCC patients. Based on the results, succinate, a key metabolite of the tricarboxylic acid cycle pathway, was accumulated in the primary cultured precancerous OLP keratinocytes and OSCC cells. Then, we found that succinate activated the hypoxia‐inducible factor‐1 alpha (HIF‐1α) pathway and induced apoptosis, which could also be up‐regulated by the tumour suppressor lncRNA MEG3. These results suggested the critical roles of succinate and MEG3 in the metabolic changes during malignant transformation from OLP to OSCC, which indicated that succinate, HIF1α and downstream proteins might serve as new biomarkers of precancerous OLP for early diagnosis and therapeutic monitoring. In addition, succinate or its prodrugs might become a potential therapy for the prevention or treatment of OSCC.

## 1 INTRODUCTION

Oral squamous cell carcinoma (OSCC) holds 95% of all types of head and neck cancer, and the morbidity increased 50% over the last decade.^1^ The primary cause of the high morbidity and low survival rate lies on that OSCC is diagnosed at late stages (stage III or IV) in most cases,^2^ which significantly impairs the patients’ quality of life. Clinical data demonstrated that oral carcinogenesis is a chronic process, in which multiple stages may take their parts, including inflammation,^3^ precancerous lesions, invasion and metastasis.^4^ Among the precancerous lesions of OSCC, oral lichen planus (OLP) is one of the frequently happening chronic mucocutaneous inflammatory diseases. Thus, for early diagnosis and precise treatment of OSCC, the mechanism underlying malignant transformation from OLP to OSCC should be fully investigated.^5^, ^6^

In recent years, the concept of precision medicine leads cancer treatment to a new era. Research on tumour mechanisms and classifications have advanced to a particular pathway or molecular level. Several studies revealed possible molecular mechanisms or biomarkers of OLP malignant transformation from different aspects. Nosratzehi et al^7^ evaluated four types of salivary matrix metalloproteinases (MMPs) obtained from OLP, OSCC patients and healthy controls, and reported higher levels of MMP‐2 and MMP‐13 in OSCC than in OLP and controls. Similarly, Cuevas‐Nunez et al^8^ found that 5‐hydroxylmethylcytosine may serve as a biomarker to distinguish oral epithelial dysplasia (OED) and OSCC by studying the mucosa samples from 9 normal people, 10 OLP, 15 OED and 23 OSCC patients. Yang et al^5^ assessed genome‐wide transcriptional profiles of OLP and OSCC by high‐throughput sequencing and enrichment analyses of mRNAs and long noncoding RNAs (lncRNAs), which turned out that keratinization and MHC class I antigen processing were activated during the malignant transformation. Another carcinogenesis genome‐wide analysis by Tang et al^9^ indicated decreased expression of metabolic pathways in OSCC, such as the tricarboxylic acid (TCA cycle).

Plenty of evidence suggest that malignant transformation closely relates to changes in several branches of metabolism (eg TAC).^10^ In addition, the accumulation of metabolites such as succinate (SUC), fumaric acid (FUM) and 2‐hydroxyglutarate, are associated with the oncogenesis.^11^ Although genome and metabolome analyses^9^, ^12^, ^13^ shed light on specific metabolic pathways and enzymes changes between OSCC, OLP and healthy control samples, the molecular profile of changes in TAC is still not clear enough for the intervention of OSCC, and the lack of proper biomarkers on this pathway hinders the diagnosis of malignant transformation from OLP to OSCC.

In this research project, we first evaluated the content changes in several important metabolites of TAC during malignant transformation by examining the innovatively primary cultured OLP and OSCC cell lines from patients. Then, we investigated the effects of SUC (a key metabolite of TAC) on the hypoxia‐inducible factor‐1 alpha (HIF‐1α), which could be activated by prolyl hydroxylase (PHD) inhibition.^14^ The accumulation of SUC was found to promote apoptosis by activating the HIF‐1α pathway and could be induced by lncRNA 01 MEG3. Finally, the changes in succinate‐HIF‐1α pathway were validated on clinical samples. Our study suggested that the MEG3‐SUC‐HIF‐1α pathway‐induced apoptosis might act as a protection against malignant transformation, which made SUC administration has a potential therapy for the prevention or treatment of OSCC. In addition, SUC, HIF‐1α and downstream proteins could serve as new biomarkers for the diagnosis of OLP malignant transformation.

## 2 MATERIALS AND METHODS

### 2.1 Patients and tissue samples

In total, 30 OLP and 41 OSCC specimens were collected for study which were provided by Huashan Hospital, Fudan University from 2014 to 2017 (Table 1). Prior patient's consents and approval from the Institutional Research Ethics Committee were obtained to use the clinical samples for research purposes.

**Table 1:** Clinical characteristics of patients

Patient ID	Gender	Age	Site of OLP lesions	Type of OLP lesions	Follow‐up (mo)	Pathological number	Associated condition	HE	Primary site of OSCC
1	F	49	BM, LU	II	24	X15‐02444	None	OLP	
2	F	68	BM‐R	I	36	X15‐02275	Hypertension, diabetes	OLP	
3	M	48	BM, PA	II	1	X15‐10742	None	OLP	
4	F	54	BM	II	12		None	OLP	
5	M	52	T, BM	I	84	X15‐04591	None	OLP	
6	F	49	BM	II	12		None	OLP	
7	M	40	BM, LU	I hyperplasia	24	X15‐04758	None	OLP‐OSCC	BM
8	F	82	T‐L	II	2	X15‐04813	Hypertension, diabetes	OLP‐OSCC	T‐L
9	M	82	BM, G, L	I and II	84		Hypertension	OLP‐OSCC	G
10	M	67	LU	I and II	60	X17‐00197	None	OLP‐OSCC	LU
11	F	68	T‐L	I	48	X16‐06256	Diabetes	OLP	
12	F	82	BM‐L	II	3		None	OLP	
13	F	66	BM‐R	I	6	X16‐00041	None	OLP	
14	F	39	T‐R	I	2	X15‐04077	None	OLP	
15	F	50	BM	II	24	X15‐06865	None	OLP	
16	M	47	BM‐L	I	6	X15‐08849	Heart disease	OLP	
17	F	64	T‐L, MF	II	24	X15‐10394	Hypertension	OLP	
18	F	66	BM‐R	I	6	X16‐00041	None	OLP	
19	F	68	T‐L	I	48	X16‐06256	Diabetes	OLP	
20	F	60	BM	I	6	X16‐07109	None	OLP	
21	F	62	BM	I	6	X16‐07152	None	OLP	
22	M	19	BM	I	2	X16‐07151	None	OLP	
23	F	39	BM	I	1	X16‐07334	None	OLP	
24	F	42	BM	II	12	X16‐07605	None	OLP	
25	F	59	BM	II	6	X16‐07750	None	OLP	
26	F	66	BM	II	12	X16‐08214	Hypertension	OLP	
27	M	70	LU	II	6	X17‐08763	Hypertension	OLP	
28	M	66	BM, LU	II	24	X16‐01643	None	OLP	
29	M	57	BM‐R	I	6	X15‐07085	Hypertension	OLP	
30	M	57	LU	I	84	X15‐10345	Diabetes	OLP	

Abbreviation: BM, buccal mucosa; G, gingiva; LU, under of lip; MF, floor of the mouth; OSCC, oral squamous cell carcinoma; PA, palatal arch; T, tongue.

### 2.2 Cells and culture

The reticular‐type lesion tissue, about 0.6 × 1.0 cm, was obtained from OLP patients by sterile technique, and immersed in sterile phosphate‐buffered saline (PBS) containing 100 units/mL penicillin and 100 mg/mL streptomycin (Gibco, USA) to remove blood. Afterwards, OLP type I keratinocytes were cultured in vitro in KSF‐M medium. Viable third‐ to fourth‐generation keratinocytes were used in this present study. Cal27 were obtained from Ninth People's Hospital, Shanghai Jiaotong University as a gift. All cells were cultured in DMEM medium (Invitrogen, USA) with 10% foetal bovine serum (HyClone, USA) at 37°C in a 5% CO_2_ atmosphere. For transfection analysis, OLP type I cells were plated on six‐well plates and transfected using Lipofectamine 2000 for 0.75 μL/well (Invitrogen, USA) with plasmid MEG3. Empty pcDNA3.1 vector was transfected as a control.

### 2.3 Identification of TAC metabolites by HPLC‐ESI‐QqQ‐MS

A high‐performance liquid chromatography (HPLC) system equipped with a diode array detector (DAD) was used to analyse samples. TAC metabolites were achieved on a Phenomenex P/N 00B‐4378‐B07 Luna NH2 (50 × 2.0 mm) (Agilent, USA). The column was thermostatically controlled at 30°C. The flow rate was set to 1 mL/min and the injection volume was 5 μL. The mobile phase consisted of two solvents: ammonium acetate (A, 100%) and acetonitrile (B, 100%). The solvent gradient in volumetric ratios was set as follows: 70%‐75% in 15 minutes; at 45 minutes, the gradient was increased to 100% A; and held at 100% A for an additional 2 minutes. The UV absorbance of the peaks were collected between 200 and 620 nm using a DAD and monitored at wavelengths of 360 nm. A triple quadrupole mass spectrometer (6430 QqQ LC/MS system; Agilent Technologies, USA) equipped with an orthogonal electrosprayionization (ESI) source were used to identify the derivatives. A negative ion mode was selected for data collection^15^, ^16^. Mass spectrometr (MS) parameters were set as follows: the sheath gas was nitrogen and collision gas was helium; drying gas flow rate, 11.0 L/min; drying gas temperature, 300°C; nebulizing gas pressure, 15 psi; the capillary voltage, 4 kV. Peak areas in HPLC chromatograms were converted into mass using the calibration curve of pure standard as the method reported.

### 2.4 Western blot analysis

Protein was extracted from cells using cell lysis solutions containing protease inhibitors and phosphorylase inhibitors. Equal amounts of protein were fractionated on Tris‐glycine SDS‐polyacrylamide gels and subjected to electrophoresis and transferred to NC membranes. Membranes were blocked with 5% non‐fat milk with Tris‐buffered saline‐Tween 20 (TBS‐T), and then incubated with primary antibodies (α‐ketoglutarate dehydrogenase (OGDH; ab137773), HIF‐1α (ab51608), BAX (ab77566), citrate synthase (CS; ab129095), VEGF (ab53465), Bcl‐2 (ab32124), isocitrate dehydrogenase (IDH; ab58641), MMP‐9 (ab194316), caspase 3 (ab32351), goat anti‐rabbit HRP (ab6721), goat antimouse HRP (ab97023); Abcam Inc Cambridge, MA). After washing in TBS‐T, membranes were incubated with fluorescent secondary antibodies. β‐Actin was used as the loading control. The signal intensity of primary antibody binding was quantitatively analysed with ImageJ software (WS Rasband, ImageJ, NIH, Bethesda, MD).

### 2.5 RNA isolation and quantitative reverse transcription‐PCR

Total RNA was extracted using Trizol reagent (Invitrogen, NY) according to the manufacturer's instructions from the control group. The primer sequences are shown in following Table 1. cDNA was prepared from 1 mg total RNA using a cDNA synthesis kit (TAKARA, Madison, WI). Quantitative reverse transcription‐PCR (qRT‐PCR) was carried out with SYBR Supermix (TAKARA). Primers used for amplification are listed in Table 2. The amplification cycle consisted of 30 seconds at 95°C, 5 seconds at 95°C, 5 seconds at 95°C and 1 second at 60°C for 40 times. The expression of each target mRNA relative to actin was calculated based on the threshold cycle (CT) as r = 2^−Δ (ΔCT)^. In addition, we commissioned Huada Gene to test microRNAs and the primers were list in the Table 1.

**Table 2:** Primers

Name	Sequence
OGDH‐F	GGCTTCCCAGACTGTTAAGAC
OGDH‐R	GCAGAATAGCACCGAATCTGTTG
IDH‐F	AGAAGCATAATGTTGGCGTCA
IDH‐R	CGTATGGTGCCATTTGGTGATT
CS‐F	GGTGGCATGAGAGGCATGAA
CS‐R	TAGCCTTGGGTAGCAGTTTCT
miR‐7‐5p‐RT	GTCGTATCCAGTGCGTGTCGTGGAGTCGGCAA TTGCACTGGATACGACAACAACA
miR‐7‐5p‐F	GTGGAAGACTAGTGATTTTG
miR‐361‐5p‐RT	GTCGTATCCAGTGCGTGTCGTGGAGTCGGCAA TTGCACTGGATACGACGTACCC
miR‐361‐5p‐F	TTATCAGAATCTCCAGGGG
U6‐RT	GTCGTATCCAGTGCGTGTCGTGGAGTCGGCAATTGC ACTGGATACGACAAAATATG
U6‐F	TTAGCATGGCCCCTGCG
miRNA primer	TGCGTGTCGTGGAGTC

### 2.6 Immunohistochemical assay

Tissues were washed with PBS for 5 minutes × 3 times. Tissues were then treated with precooled alcohol for 15 minutes before blocked in PBS containing 1% BSA and 0.1% Triton X‐100 for 1 hour at room temperature. Then, the tissues were incubated with primary antibody overnight at 4°C. After washing three times with PBS for 10 minutes, the tissues were incubated with HIF‐1α (ab51508, 1:100), IDH (ab58641, 1:100), VEGF (ab53465, 1:100), CS (ab96600, 1:200), MMP‐9 (ab194316,1:1000), OGDH (ab137773,1:100), anti‐rabbit IgG for 1 hour at room temperature. After another 10 minutes × 3 times of washing with PBS, tissues were incubated with haematoxylin for 10 minutes, and dehydrated in increasing grades of ethanol and cover‐slipped with Fluoromount Aqueous Mounting Medium (Sigma, F4680; Sant Louis, MO). The slices were analysed with a laser scanning confocal unit (Zeiss LSM 710, Carl Zeiss, Jena, Germany).

### 2.7 Cell survival and mitochondrial membrane potential assays

Cell survival assay and mitochondrial membrane potential assays were performed using the Cell Titer‐Glo Luminescent Cell Viability Assay kit (Promega, Wisconsin) and Mitochondrial membrane potential assay kit with JC‐1 (Beyotime, China). All the analyses were performed according to the manufacturer's instructions. Luminescence and fluorescence were recorded with a Biotek Synergy2 Luminescent plate reader.

### 2.8 Statistical analysis

All data were presented as means ± SEM. Data were subjected to one‐way ANOVA using the GraphPad Prism software statistical package (GraphPad Software). When a significant group effect was found, post hoc comparisons were performed with the Newman‐Keulst test to examine special group differences. Independent group *t* tests were used for comparing two groups. Significant differences with *P* < 0.05, *P* < 0.01, and *P* < 0.001 are indicated by *, **, ***, respectively. All calculations were performed with the 14.0 spss software package (SPSS Inc).

## 3 RESULTS

### 3.1 TAC was suppressed in the process of carcinogenesis

**Figure 1:**
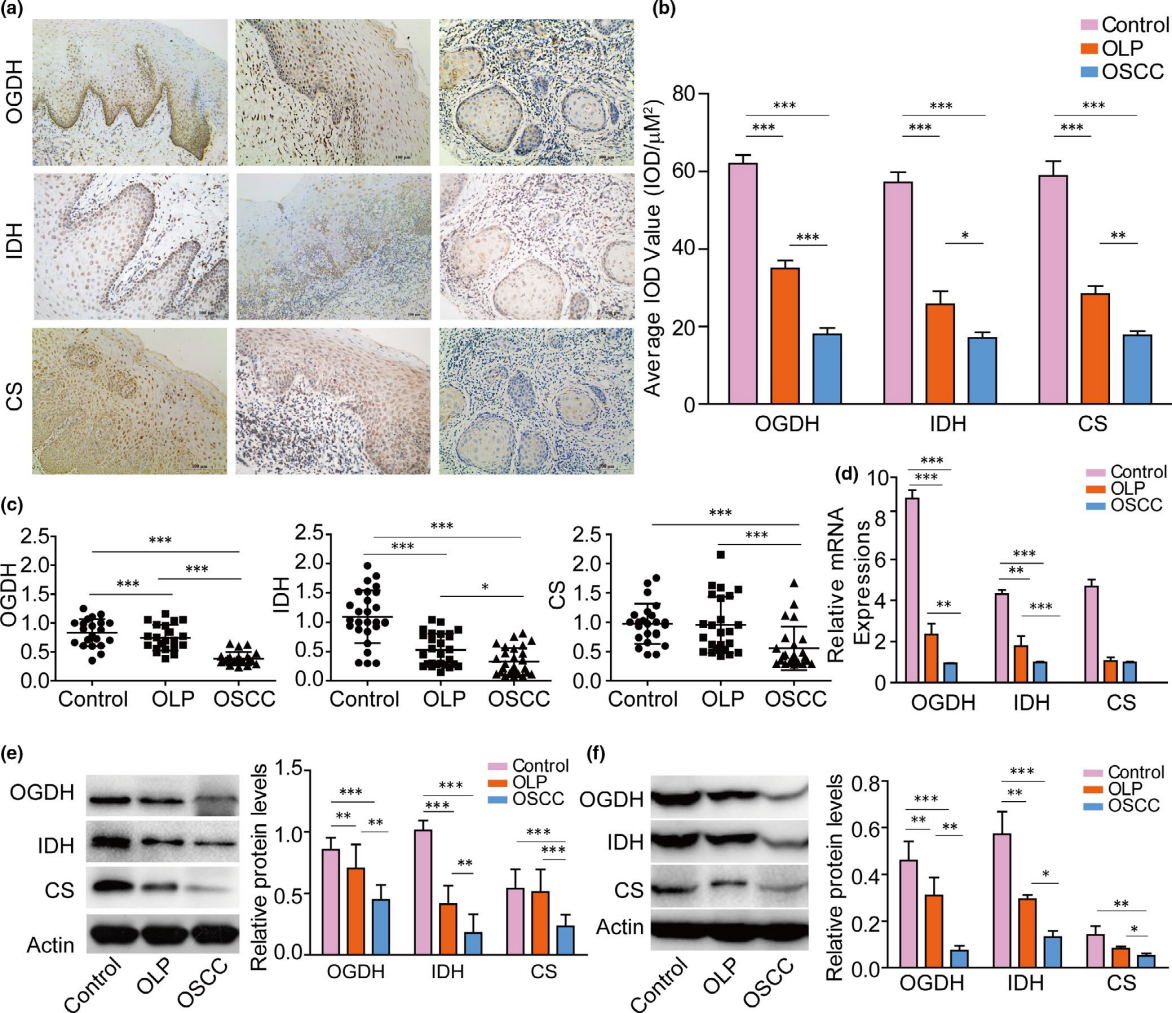
Down‐regulated expression of α‐ketoglutarate dehydrogenase (OGDH), isocitrate dehydrogenase (IDH) and citrate synthase (CS) in oral lichen planus (OLP) and oral squamous cell carcinoma (OSCC). (A), immunohistochemistry staining localization of OGDH, IDH and CS in the oral mucosa from normal oral mucosa (NOM) (left), OLP (middle) and OSCC (right) samples. Enzymes, yellow; magnification, 200×. (B), Semi‐quantitative analysis demonstrated the reduced expression levels of OGDH, IDH and CS in OLP and OSCC compared with NOM, and significantly lower expression of the three enzymes in OSCC tissue than OLP. Quantitative reverse transcription‐PCR experiment showed similar results for OGDH (left), IDH (middle) and CS (right) in NOM, OLP and OSCC at the tissue level (C) or cellular level (D). Western blot results were consistent with B, C and D at the tissue level (E) or cellular level (F). *t* test: *P < 0.05; **P < 0.01; ***P < 0.001

Tissue samples of normal oral mucosa (NOM), OLP mucosa and OSCC nests were collected from healthy volunteers and patients. Immunohistochemistry was performed to evaluate the localization and the expression of OGDH, IDH and CS. As shown in Figure 1A, all the three enzymes were detectable in NOM, OLP and OSCC tissues and mainly located in the cytoplasm. However, according to the immunohistochemistry semi‐quantitative analysis, the IOD values of all three enzymes were significantly decreased in OLP and OSCC tissues compared with NOM. In addition, a lower expression of OGDH, IDH and CS was also observed in OSCC than OLP (Figure 1B). To confirm their down‐regulation behaviours, qRT‐PCR and Western blot experiments were conducted. Again, lower expressions of OGDH, IDH and CS were observed in OLP tissues compared with NOM, and their expression decreased even more in OSCC tissues (Figure 1C and 1). Similar results were obtained at the cellular level (Figure 1D and 1). These results implied that with the development from OLP to OSCC, the levels of key enzymes in TAC were gradually down‐regulated. Dysfunction of energy metabolism was involved in the process of malignant transformation. Not only OSCC cell line, OLP keratinocytes also exhibited lower level of oxidative phosphorylation, and could be taken as precancerous cells for the following cytology experiments to study the properties of malignant transformation from OLP to OSCC. To our knowledge, this was the first primary cultured OLP precancerous cell line in China.

### 3.2 Succinate was accumulated for OLP and OSCC at both the tissue and cellular level

**Figure 2:**
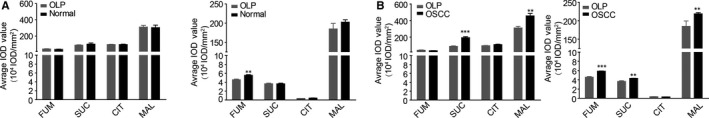
Succinate was accumulated in precancerous oral lichen planus (OLP) keratinocytes (A) no obvious change in the expression of TAC metabolites were observed in OLP versus normal oral mucosa at tissular level (left), but the expression of fumaric acid (FUM) was down‐regulated in OLP at the cellular level (right). (B), The expressions of citric acid (CIT), succinate (SUC), malic acid (MAL) were up‐regulated in oral squamous cell carcinoma (OSCC) compared with OLP at the tissue level (left), while the levels of SUC and FUM were higher in OSCC than OLP at the cellular level (right)

The suppression of energy metabolism might change the level of its metabolites, which could be essential in the process of carcinogenesis. We examined several key metabolites of TAC, including citric acid (CIT), SUC, FUM and malic acid (MAL), by HPLC‐MS There was no significant difference in the expression levels of these four kinds of metabolites between normal and OLP tissues. However, loss of FUM was observed in OLP keratinocytes compared with normal cells (Figure 2A). In contrast, CIT, SUC and MAL were up‐regulated in OSCC compared with OLP tissues. But in the cellular level, SUC and FUM were increased in OSCC (Figure 2B). Taken these together, SUC was accumulated in both OSCC tissues and cells, which suggested that SUC might closely relate with the malignant transformation from OLP to OSCC.

### 3.3 Succinate‐HIF‐1α pathway activation could suppress cell proliferation and promote apoptosis of OLP precancerous cells

**Figure 3:**
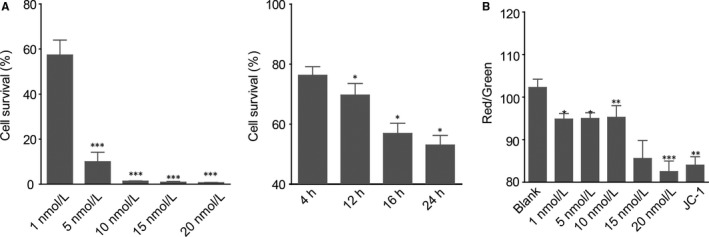
The apoptosis effect of succinate (SUC) on oral lichen planus keratinocytes. (A), Cell viability under different concentrations of SUC at different time points measured by ATP ELISA. (B), SUC significantly decreased the mitochondrial membrane potential with dose dependency. JC‐1, positive control

ATP ELISA was employed to test the cell viability under SUC accumulation. Stimulating OLP cells for 24 hours, 1 mmol/L of SUC reduced the survival rate to below 60%, which continued to drop until about 10% at 5 mmol/L. The proliferation was totally inhibited at 10 mmol/L or higher. This antiproliferation effect became significant by treatment of 10 mmol/L SUC for more than 12 hours (Figure 3A). The decrease in mitochondrial membrane potential is a marker of early apoptosis. Thus, we examined the changes in mitochondrial membrane potential after treating OLP cells with SUC at different concentrations. mitochondrial membrane potential was significantly decreased by 1 mmol/L SUC. With the increase in SUC concentration, mitochondrial membrane potential decreased in a dose‐dependent manner (Figure 3B). Flow cytometry results suggested that SUC could also reduce the amount of OLP cells by late apoptosis, which also exhibited dose and time dependency (Figure S1). These results indicated that apoptosis was induced by SUC accumulation.

### 3.4 The accumulation of succinate was pivotal during malignant transformation by activating HIF‐1α pathway

**Figure 4:**
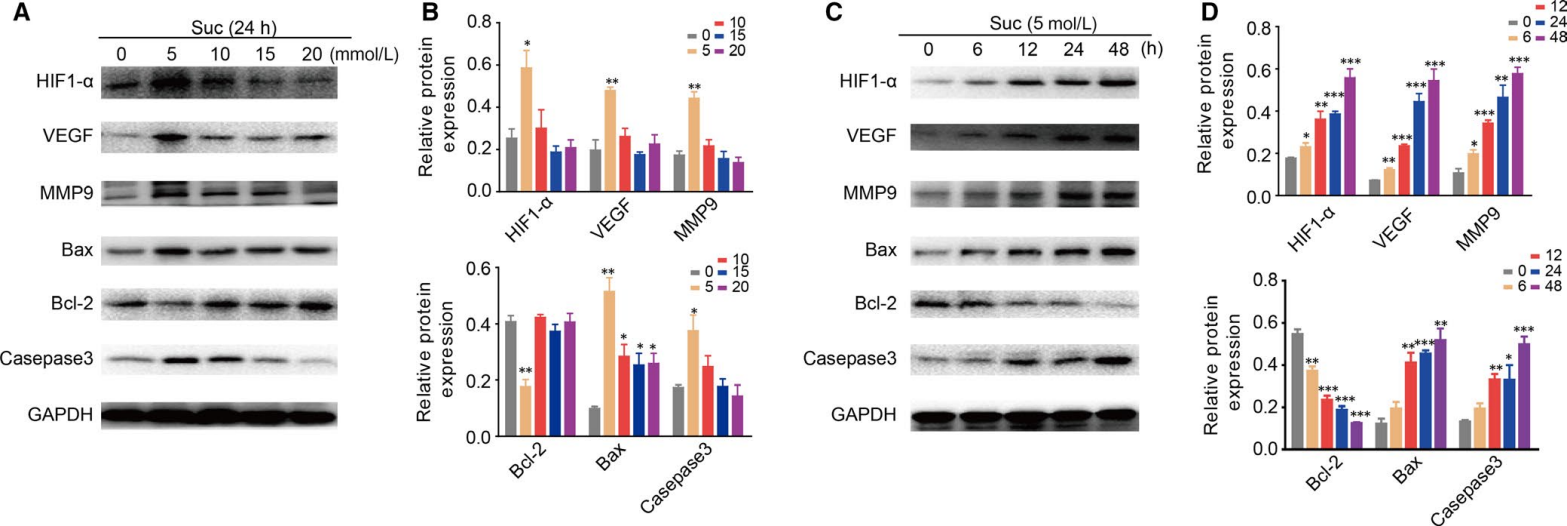
Succinate (SUC)‐activated HIF‐1α pathway. (A, B), Western blot experiments demonstrated that SUC activated HIF‐1α pathway with different doses; its downstream target proteins VEGF, MMP‐9, Bax and caspase 3 were up‐regulated, Bcl‐2 was down‐regulated. (C, D), Similar results were observed at different time points. The best dose and time were 5 mmol/L, 24 h. *t* test: *P < 0.05; **P < 0.01; ***P < 0.001

It has been reported that SUC acts as an inflammatory signal to induce the activation of HIF‐1α,^14^ because excess SUC could impair the PHD activity (by product inhibition) leading to HIF‐1α stabilization. To verify the hypothesis that SUC accumulation might activate the HIF‐1α pathway during the malignant transformation, we performed Western blot experiments to evaluate the expressions of HIF‐1α and its downstream target proteins (ie VEGF, MMP‐9, Bax, Bcl‐2 and caspase 3) in the OLP keratinocytes incubated with SUC from 5 to 20 mmol/L for different time periods ranging from 0 to 48 hours. As shown in Figure 4A,B, 5 mmol/L of SUC caused the strongest up‐regulation of the HIF‐1α pathway. Its highest expression level was achieved at the 24th hour (Figure 4C,D). The promotion of HIF‐1α pathway exhibited dose dependency and time dependency when stimulated with SUC, which suggested that SUC accumulation was able to up‐regulate HIF‐1α and its downstream proteins.

Interestingly, after incubating with SUC at a proper dose for a proper time period, all these regulated proteins were increased (ie HIF‐1α, VEGF, MMP‐9, Bax and caspase 3) except Bcl‐2. This result accorded with their effect on the apoptosis, as HIF‐1α, VEGF, MMP, Bax and caspase were reported to promote apoptosis, while Bcl‐2 could suppress it.^17^, ^18^, ^19^, ^20^ Thus, the SUC accumulation might also tend to launch apoptosis during malignant transformation from OLP to OSCC.

### 3.5 LncRNA MEG3/miR‐361‐5p/SDHD pathway could increase the content of succinate and lead to cell apoptosis

**Figure 5:**
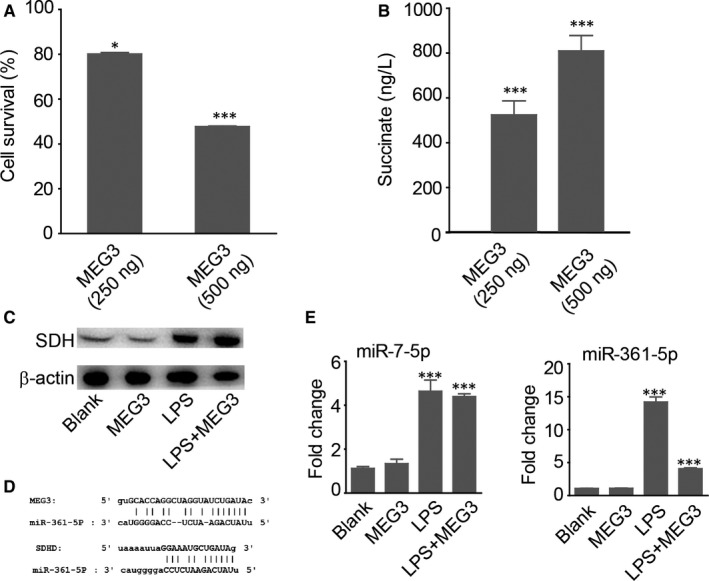
Long noncoding RNA (LncRNA) MEG3/miR‐361‐5p/SDHD pathway‐modified apoptosis. (A, B), After treatment with lncRNA MEG3, the cell survival rate was decreased and succinate (SUC) was accumulated, both in a dose‐dependent way. *t* test: *P < 0.05; **P < 0.01; ***P < 0.001. C, Western blot experiments demonstrated that SUC increased. (D, F), Bioinformatics predicted miR‐361‐5P binding site with MEG3 and SDHD

Long noncoding RNA MEG3 has been reported to be a tumour suppressor.^21^ It is also involved in regulating glycolysis in the tumours and serves as a novel biomarker and pharmaceutical target. To further confirm the apoptosis promotional effect of SUC accumulation in precancerous OLP cells, we evaluated the expression of SUC after incubating OLP keratinocytes with lncRNA MEG3. As shown in Figure 5A, 500 ng MEG3 caused apoptosis of more than half of the precancerous cells. Meanwhile, the concentration of SUC was increased to about 800 ng/L, seven times higher than the blank control (Figure 5B). The survival rate and SUC increase also exhibited dose dependency, which indicated strong association between SUC accumulation and MEG3‐induced apoptosis of malignant cells. Succinate dehydrogenase (SDH) is an enzyme complex, which catalyse the accumulation of SUC (Figure 5C). Increasing research have confirmed that lncRNAs may be function as a molecular sponge or a ceRNA via competitively binding to miRNA leading to the variation in its targeted mRNA in regulating tumour development and pathogenesis. To explore whether MEG3 had the similar function to regulate certain miRNAs, Starbase was used to predict potential miRNAs that directly interacted with MEG3 and SDH (supplementary table). We found miR‐7‐5p and miR‐361‐5p were the most potential targets as both interacted with MEG3 and SDH (Figure 5D). Furthermore, qRT‐PCR showed that miR‐361‐5p was significantly down‐regulated upon MEG3 stimulation, while miR‐7‐5p remained unchanged (Figure 5E). Thus, SUC accumulation and HIF‐1α activation‐induced apoptosis could be a downstream effect of MEG3.

### 3.6 Validation of succinate, HIF‐1α, VEGF and MMP‐9 levels on clinical samples

**Figure 6:**
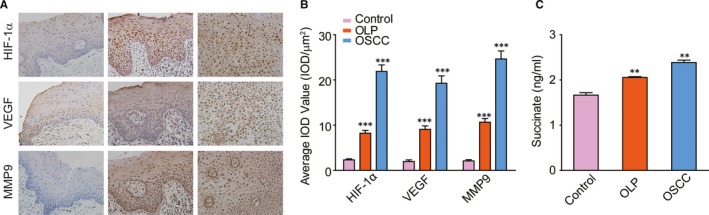
Clinical validation of the expression changes in succinate (SUC), HIF‐1α, VEGF and MMP‐9 on normal oral mucosa (NOM), oral lichen planus (OLP) and oral squamous cell carcinoma (OSCC) tissue samples. (A), Immunohistochemistry staining localization of HIF‐1α, VEGF and MMP‐9 in the oral mucosa from NOM (left), OLP (middle) and OSCC (right) samples. Enzymes, yellow; magnification, 200×. (B), Semi‐quantitative analysis demonstrated significant up‐regulation of HIF‐1α, VEGF and MMP‐9 in OSCC compared with OLP, also significant in OLP compared with NOM. (C), SUC was accumulated in the OLP tissue, and even more in the OSCC nests. *t* test: *P < 0.05; **P < 0.01; ***P < 0.001

Additional NOM, OLP mucosa and OSCC nests (10 samples each) were collected to validate the changes in the SUC and HIF‐1α pathway. A semi‐quantitative analysis of the immunohistochemistry experiment demonstrated that HIF‐1α, VEGF and MMP‐9 were up‐regulated in OLP tissue compared with NOM, and further increased in OSCC tissue (Figure 6A,B). Succinate ELISA result showed significant accumulation of SUC in OLP tissue (about twice of that in NOM), and also more SUC was observed in OSCC nests (Figure 6C).

## 4 DISCUSSION

In this work, we found SUC, an important metabolite of TAC, played pivotal roles in the malignant transformation from OLP to OSCC. To our knowledge, this is the first study that confirm the importance of SUC on primary OLP precancerous cells from Chinese patients. The level of oxidative phosphorylation was gradually reduced in NOM, OLP and OSCC, which caused variations in the levels of different metabolites. Among them, the most profound one was SUC, which was accumulated in OLP precancerous lesion, and more preserved in OSCC cells. The accumulation of SUC could activate HIF‐1α in a dose‐ and time‐dependent manner. Interestingly, the changes in downstream target proteins of HIF‐1α are in accordance with their effect on apoptosis. Thus, we could infer that SUC accumulation was helpful for the apoptosis of precancerous cells. After ATP ELISA test, it turned out that SUC was indeed able to induce apoptosis at higher level in precancerous OLP cells. In addition, to find out if SUC accumulation is a key point of apoptosis promotion, we measured the fold change in SUC after treating OLP cells with the tumour suppressor lncRNA MEG3. The apoptosis induced by MEG3 was also accompanied by SUC accumulation, indicating a mechanism of tumour suppression by MEG3 might be through SUC accumulation, HIF‐1α activation and apoptosis promotion during the malignant transformation from OLP to OSCC. These results were validated on additional clinical samples, in which the expression levels of SUC, HIF‐1α, VEGF and MMP‐9 were gradually up‐regulated in NOM, OLP and OSCC. Our study demonstrated the important roles of the protective metabolism changes and their relationship with the tumour suppressor lncRNA MEG3 during the OLP malignant transformation process. This work suggested that the succinate‐HIF‐1α pathway might serve as biomarkers for the diagnosis of precancerous OLP. Also, SUC might be helpful in the prevention and treatment of OSCC.

More than 90% of all cancer cases are related with chronic infection or other inflammatory environments, which affect almost all stages of cancer development.^22^ However, early‐stage cancer and precancerous inflammation are usually ignored or treated improperly, causing the loss of best chances for saving lives. Precise medical projects might mend the traditional cancer diagnosis, treatment and prognosis by providing the patient precise or even personalized therapy according to one's examinations on different biomarkers. Cancer biomarkers are defined as biological molecules found in blood, saliva, or other body fluids or tissues that are signs to distinguish cancerous condition from normal status.^23^ For example, l‐phenylalanine in saliva is one of the biomarkers for the early diagnosis and monitoring of OSCC. Biomarkers can also be used to identify therapeutic targets. Monteiro et al^24^ suggested the highly expressed p‐mTOR is a potential drug target in OSCC patients. With the popularization of DNA‐seq, RNA‐seq and other bioinformatics technology, it would become routine to use these methods to trace the occurrence and development of different cancer types. These projects should tremendously improve the survival rates and the quality of patients’ life, on condition that the molecular mechanisms of the tumours are fully investigated and the patients were correctly divided into the specific subtype of a general cancer class, which requires hardworking of years by medical scientists. In this case, OLP might be a precancerous condition, in which immunoinflammatory processes drives the pathogenesis and malignant transformation. Meanwhile, the metabolism pathways have been reported to change during the development of carcinogenesis. Thus, the association between metabolic variation and malignant transformation from OLP to OSCC needs to be revealed. In addition, key metabolites and the downstream target proteins could also serve as new biomarkers or drug targets for the diagnosis and therapeutic monitoring of OSCC before late stages.

Our investigation of the profile of metabolic changes in TAC elucidated its strong association with the malignant transformation from OLP to OSCC. We revealed that, SUC, one of the key metabolites of TAC, would accumulate in the precancerous OLP keratinocytes and OSCC cells to induce apoptosis by activating HIF‐1α, VEGF and MMP‐9 in a dose‐ and time‐dependent manner. In addition, SUC accumulation was a downstream effect of the tumour suppressor lncRNA MEG3. Thus, for the first time to our knowledge, lncRNA MEG3‐induced apoptosis was linked to the metabolism pathway as the cause of the suppressed oxidative phosphorylation during OSCC carcinogenesis. Although for many cases, the overexpressed components were pathogenic, but according to our research, SUC accumulation acted as a role of protection by inducing the apoptosis of precancerous cells. Our body is a complicated and self‐regulating system. Other than getting rid of pathogenic factors (eg gene mutation in cancer), it is more implementable to enhance the protectors such as launching apoptosis in the dangerous cells. Our work suggested that administration of SUC or its prodrugs might prevent the advancement of early OSCC. In addition, SUC, HIF‐1α, VEGF and MMP‐9 could serve as new biomarkers for the early diagnosis and therapeutic monitoring of OSCC.

## CONFLICT OF INTERESTS

The authors declare that they have no conflict of interests.

## AUTHOR CONTRIBUTION

QZ Yang designed experiments, analysed data, conceptualized this project and constructed the manuscript. XX Wang and XD Yu designed experiments and analysed data, and provided scientific advice. J. Zhang provided clinical specimens and collected the patient's information. B. Guo and Y. Sai implemented experiments, and analysed data, provided formal analysis. HY Sun provided resources and supervision, conceptualized this project, analysed data, provided scientific advice and constructed the manuscript.
