# Supplementary material for: Metabolic changes during malignant transformation in primary cells of oral lichen planus: Succinate accumulation and tumour suppression
Source: J Cell Mol Med. 2019 Dec 2;24(2):1179–88. doi: 10.1111/jcmm.14376 (PMC6991640; doi:10.1111/jcmm.14376)
Supplement: Supplementary file 1 [file JCMM-24-1179-s001.pdf]

SUPPORTING INFORMATION

Metabolism Changes during Malignant Transformation in Primary Cells of Oral Lichen Planus: Succinate Accumulation and Tumor Suppression

Contents

Figure S1. Flow cytometry results of OLPkeratinocytes treated with SUC.

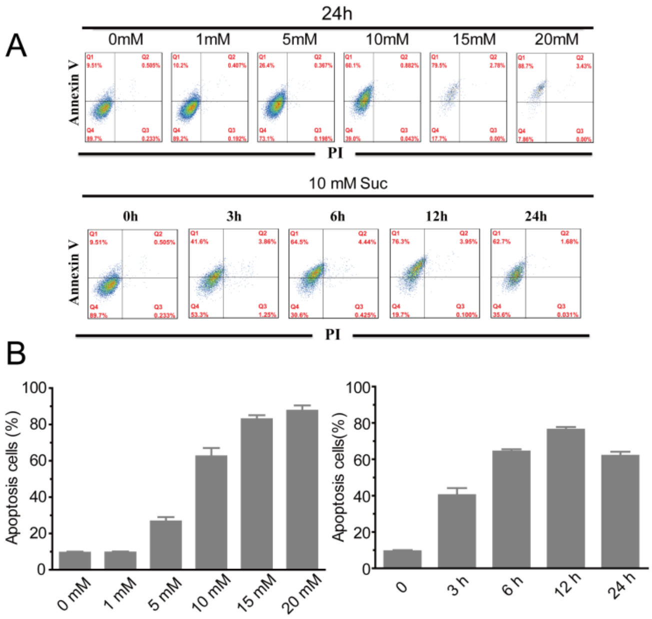

Figure S1.Flow cytometry results of OLPkeratinocytes treated with SUC. A, apoptosis was observed after treatedOLP keratinocytes with SUC. **B**, they were both in a dose- and time-dependent way.
