# Supplementary material for: Metabolic changes during malignant transformation in primary cells of oral lichen planus: Succinate accumulation and tumour suppression
Source: J Cell Mol Med. 2019 Dec 2;24(2):1179–88. doi: 10.1111/jcmm.14376 (PMC6991640; doi:10.1111/jcmm.14376)
Supplement: Supplementary file 2 [file JCMM-24-1179-s002.docx]

**Predicted target miRNA for MEG3**

| name | mirAccession | geneName | targetSites | bioComplex | clipReadNum | cancerNum |
| --- | --- | --- | --- | --- | --- | --- |
| hsa-miR-7-5p | MIMAT0000252 | MEG3 | 1 | 1 | 40 | -1 |
| hsa-miR-4782-3p | MIMAT0019945 | MEG3 | 1 | 1 | 17 | -1 |
| hsa-miR-219a-5p | MIMAT0000276 | MEG3 | 1 | 1 | 17 | -1 |
| hsa-miR-361-5p | MIMAT0000703 | MEG3 | 1 | 1 | 17 | -1 |

**Predicted target miRNA for SDHD**

| name | geneName | miRandaSites | CancerNum |
| --- | --- | --- | --- |
| hsa-miR-137 | SDHD | 1[3,18] | 4 |
| hsa-miR-320b | SDHD | 1[3,33] | 5 |
| hsa-miR-488-3p | SDHD | 1[6,62] | 2 |
| hsa-miR-210-3p | SDHD | 1[6,58] | 4 |
| hsa-miR-139-5p | SDHD | 1[4,26] | 1 |
| hsa-miR-196a-5p | SDHD | 1[4,26] | 4 |
| hsa-miR-320d | SDHD | 1[3,33] | 4 |
| hsa-miR-543 | SDHD | 1[2,2] | 4 |
| hsa-miR-376c-3p | SDHD | 1[4,41] | 4 |
| hsa-miR-376a-3p | SDHD | 1[4,41] | 4 |
| hsa-miR-376b-3p | SDHD | 1[4,41] | 3 |
| hsa-miR-539-5p | SDHD | 1[3,25] | 2 |
| hsa-miR-382-5p | SDHD | 1[6,59] | 4 |
| hsa-miR-410-3p | SDHD | 1[2,2] | 5 |
| hsa-miR-7-5p | SDHD | 1[1,1] | 5 |
| hsa-miR-320c | SDHD | 1[3,33] | 6 |
| hsa-miR-23a-3p | SDHD | 1[4,41] | 5 |
| hsa-miR-216a-5p | SDHD | 1[5,313] | 0 |
| hsa-miR-216b-5p | SDHD | 1[5,313] | 2 |
| hsa-miR-28-5p | SDHD | 1[3,25] | 2 |
| hsa-miR-874-3p | SDHD | 1[1,8] | 2 |
| hsa-miR-196b-5p | SDHD | 1[4,26] | 3 |
| hsa-miR-590-3p | SDHD | 2[2,2] | 2 |
| hsa-miR-320a | SDHD | 1[3,33] | 4 |
| hsa-miR-876-5p | SDHD | 1[6,59] | 1 |
| hsa-miR-23b-3p | SDHD | 1[4,41] | 1 |
| hsa-miR-374b-5p | SDHD | 1[3,25] | 3 |
| hsa-miR-374a-5p | SDHD | 1[3,25] | 2 |
| hsa-miR-384 | SDHD | 2[7,66] | 0 |
| hsa-miR-361-5p | SDHD | 1[1,8] | 3 |
| hsa-miR-542-3p | SDHD | 1[3,295] | 7 |
| name | geneName | miRandaSites | CancerNum |
